# Mechanism study on the influences of buffer osmotic pressure on microfluidic chip-based cell electrofusion

**DOI:** 10.1063/5.0205100

**Published:** 2024-04-17

**Authors:** Mengli Xu, Xiaoling Zhang, Yaqi Bai, Xuefeng Wang, Jun Yang, Ning Hu

**Affiliations:** 1 Key Laboratory of Biorheological Science and Technology, Ministry of Education and Bioengineering College, Chongqing University, Chongqing 400044, China; 2 School of Smart Health, Chongqing College of Electronic Engineering, Chongqing 401331, China

## Abstract

Cell electrofusion is a key process in many research fields, such as genetics, immunology, and cross-breeding. The electrofusion efficiency is highly dependent on the buffer osmotic pressure properties. However, the mechanism by which the buffer osmotic pressure affects cell electrofusion has not been theoretically or numerically understood. In order to explore the mechanism, the microfluidic structure with paired arc micro-cavities was first evaluated based on the numerical analysis of the transmembrane potential and the electroporation induced on biological cells when the electrofusion was performed on this structure. Then, the numerical model was used to analyze the effect of three buffer osmotic pressures on the on-chip electrofusion in terms of membrane tension and cell size. Compared to hypertonic and isotonic buffers, hypotonic buffer not only increased the reversible electroporation area in the cell-cell contact zone by 1.7 times by inducing a higher membrane tension, but also significantly reduced the applied voltage required for cell electroporation by increasing the cell size. Finally, the microfluidic chip with arc micro-cavities was fabricated and tested for electrofusion of SP2/0 cells. The results showed that no cell fusion occurred in the hypertonic buffer. The fusion efficiency in the isotonic buffer was about 7%. In the hypotonic buffer, the fusion efficiency was about 60%, which was significantly higher compared to hypertonic and isotonic buffers. The experimental results were in good agreement with the numerical analysis results.

## I. INTRODUCTION

For cells exposed to a high intensity external electric field, the transmembrane potential (TMP) across the cell membrane increases. When the TMP exceeds a certain critical value (∼1 V), pores are formed in the cell membrane, which is called electroporation.^1,2^ Electroporation on cell membranes can be divided into reversible electroporation and irreversible electroporation due to the different intensity and duration of the applied electric field. As one of the most important applications of cell reversible electroporation, cell electrofusion mainly uses electric field to induce cell pairing, membrane reversible electroporation, membrane reconstruction, and cytoplasm exchange to form hybrid cells.^3^ Cell electrofusion is a key process in the field of genetics,^4,5^ immunology,^6,7^ and cross-breeding.^8,9^ Compared with virus-induced and PEG-induced cell fusion methods, electrofusion is of great interest because of its high efficiency, non-toxicity, wide range of applicable cell types, and simple operation.^5,10,11^ Cell pairing and reversible electroporation are the two most important steps in the electrofusion process. They are mainly influenced by the local electric field. Typically, cell pairing is achieved in a sinusoidal alternating current (AC) field of high frequency (1–3 MHz) and low intensity (100–300 V/cm). Then, a series of high intensity (1–10 kV/cm) and short duration (10–50 *μ*s) electrical pulses are applied to induce cell reversible electroporation.^12^

To improve the electrofusion efficiency, a growing number of microelectrode/microfluidic structures have been designed for cell electroporation^13,14^ and electrofusion.^15,16^ Previously, our group developed and tested a series of microelectrode arrays for cell electrofusion.^17–19^ To further optimize the electric field distribution, 3D thin film microelectrodes^20^ and discrete co-planar vertical sidewall microelectrodes^21^ were developed. Although this structure improved the electrofusion efficiency, irreversible electroporation was prone to occur at the point of contact between discrete microelectrodes and cells. On this basis, we designed micro-cavity microelectrodes,^22^ which enable reversible electroporation to occur at controllable locations on the cell membrane. Therefore, it further improves the cell electrofusion efficiency. In addition, cell electroporation and electrofusion efficiency are also highly dependent on the buffer osmotic pressure properties.^6^ Previous studies have shown that hypotonic buffer was beneficial for cell electroporation by inducing cell swelling.^23,24^ However, the details of how hypotonic buffer improves cell fusion remain unclear. Sarkar *et al.*^25^ studied the effect of buffer osmotic pressure on the rupture rate constant of giant unilamellar vesicles (GUV) and estimated the GUV membrane tension corresponding to different buffer osmotic pressures using electroporation technique. The estimated membrane tension values agreed well with theoretical calculations within experimental error. Finally, they also observed the leakage of sucrose within the GUV by inducing the formation of pores in an ultra-hypotonic buffer. However, they did not provide an explanation for how the hypotonic buffer affected GUV electroporation or how GUV electroporation varied under different buffer osmotic pressures. Hamdi *et al.*^26^ used a microfluidic structure that induced a specific electric field to study the effect of buffer osmotic pressure on the cell volume, viability, and dielectrophoretic effects. An optimal buffer (100 mOsmol/kg, 0.03 S/m) scheme that could improve the efficiency of cell electrofusion was proposed. The electrofusion efficiency was as high as 75%, and the average membrane fusion time was less than 12 s. This microfluidic platform was a powerful tool to study electric field/cell interactions in real time. They primarily focused on the experimental aspects and did not delve into the numerical studies of cell electroporation and electrofusion. In conclusion, although the idea that hypotonic buffer is helpful for cell electroporation and electrofusion has been recognized, previous studies mainly focused on experiments. There is no suitable tool to quantitatively analyze and explain why from a theoretical point of view. In this study, a microfluidic structure with two parallel main channels and a series of paired arc micro-cavities was designed, and an electroporation model describing the cell electrofusion process was developed based on the above-mentioned structure. Based on the distribution of pore density and pore radius on the cell membrane predicted by the model, the mechanism by which buffer osmotic pressure influences cell electroporation and electrofusion can be easily explored. This will provide a suitable tool to guide the design of electrofusion structure, optimize the condition of electrofusion, and improve the efficiency of cell electroporation in electrofusion and other applications.

## II. RESULTS AND DISCUSSION

### A. Electroporation in cell electrofusion

**FIG. 1.**
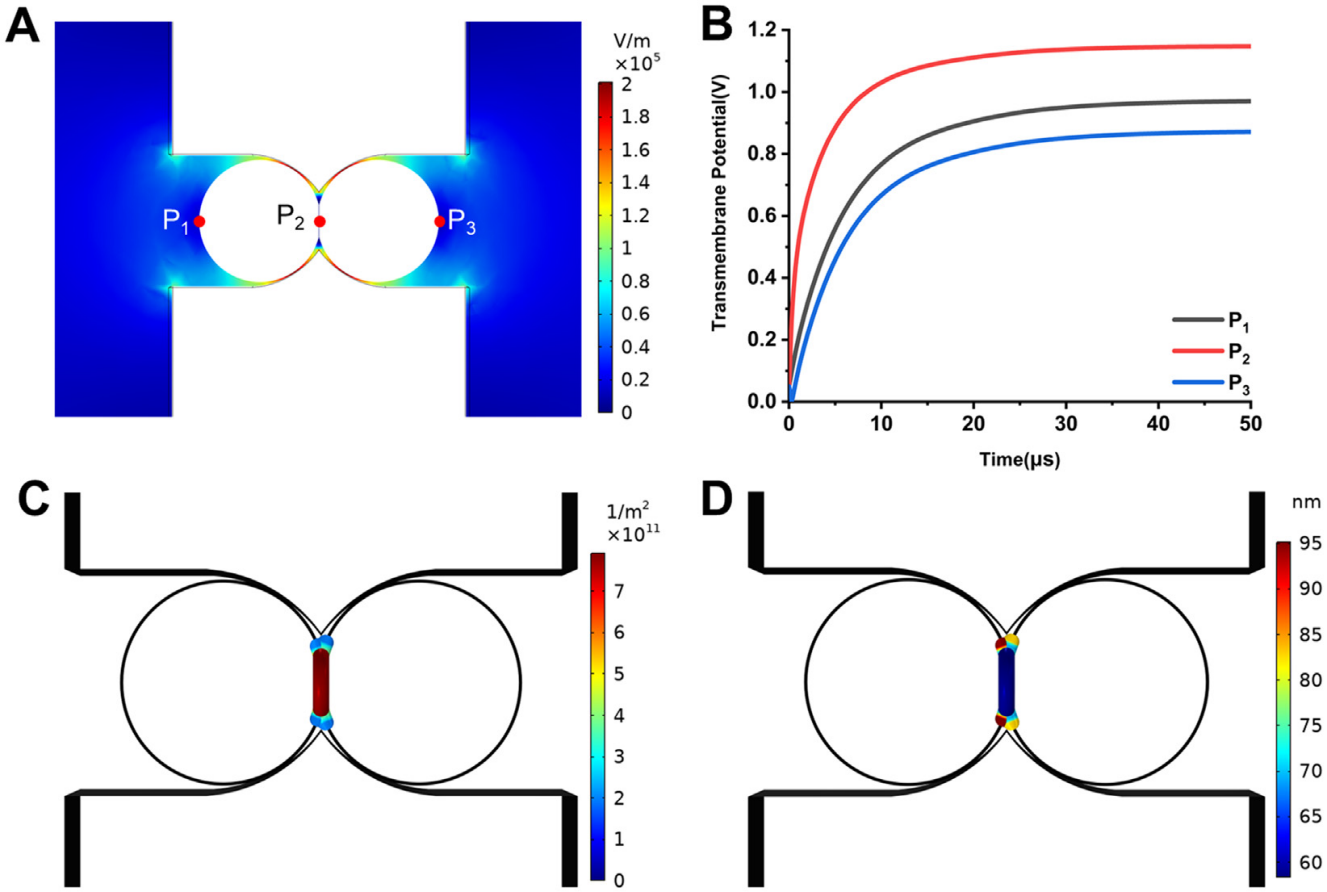
(a) Spatial distribution of electric field intensity in microchannels when cells are paired; (b) temporal variation of absolute value of transmembrane voltage at P_1_, P_2_, and P_3_; (c) pore density distribution on the cell membrane; and (d) the distribution of pore radius on the cell membrane.

The distributions of TMP and pores are highly related to the spatial distribution of the electric field, which is decided by the structure of the microchannel. Figure 1(a) depicts the spatial distribution of the electric field intensity in the microchannel when cells are paired. The electric field is concentrated within the arc micro-cavities, and the electric field intensity is highest near the cell–cell contact zone (P_2_), which is 2.01 × 10^5^ V/m. The electric field intensity is weaker at the cell poles (P_1_ and P_3_). This distribution of electric field intensity causes the TMP of P_2_ to reach steady state faster than that of P_1_ and P_3_ [Fig. 1(b)], and the TMP of P_2_ is always higher than that of P_1_ and P_3_. After reaching steady state, the TMP of P_2_ is 1.15 V, and the TMP of P_1_ and P_3_ never reaches the threshold voltage of 1 V for electroporation. This allows the resulting pores to be concentrated in the cell–cell contact zone near P_2_. Figures 1(c) and 1(d) show the pore density and pore radius distribution on the cell membrane, respectively. Membranes with pore density between 1/10 maximum and maximum were marked with a colored legend.^22^ The two cells produce pores only in the cell–cell contact zone with a pore density of up to 7.91 × 10^11^ m^−2^ and moderate pore size with a radius of about 60 nm. Very few pores are produced at the edge of the cell–cell contact zone, but the largest pore is generated with a pore size of 95 nm. The membranes near P_1_ and P_3_ do not produce pores. These results suggest the microfluidic structure with arc micro-cavities can selectively control the location of electroporation in the cell–cell contact zone, which is ideal for cell electrofusion. This is because the above-mentioned pore distribution facilitates subsequent cytoplasm exchange and membrane reconstruction during electrofusion and prevents cell rupture from occurring near P_1_ and P_3_, which increases the probability of cell electrofusion.

Although the two cells are located in symmetrical positions in the microchannel, their TMP [Fig. 1(b)] and pore distribution [Figs. 1(c) and 1(d)] are asymmetric. The TMP at the left cell pole (P_1_) is slightly larger than that at the right cell pole (P_3_) by 0.1 V. The pores in the cell–cell contact zone of the right cell are slightly more than those in the cell–cell contact zone of the left cell, and the pore radius of the former is slightly smaller than that of the latter. This is due to the effect of the resting potential. The resting potential is an intrinsic property of the cell and is usually between −90 and −40 mV. When considering the resting potential, the electric field intensity of the cell membrane facing the anode side (the P_1_ point of the left cell and the cell–cell contact zone of the right cell) is |*E*_2_| (applied electric field) + |*E*_1_| (resting electric field). The electric field intensity of the cell membrane facing the cathode side (point P_3_ of the right cell and the cell–cell contact zone of the left cell) is |*E*_2_| − |*E*_1_|. Obviously, here |*E*_2_| + |*E*_1_| > |*E*_2_| − |*E*_1_|. This asymmetric distribution of the electric field results in an asymmetry in the TMP, which in turn leads to the asymmetric distribution of pores induced by electroporation. The above-mentioned asymmetry was also mentioned by Tekle *et al.*^27^ They found in electroporation experiments that small molecule probes (calcium ion and ethidium bromide) preferentially cross the cell membrane facing the anode side, and macromolecular probes (propidium iodide and homodimeric ethidium) preferentially cross the cell membrane facing the cathode side. They believed this is due to the creation of asymmetric pores on both sides of the membrane. The pore size on the anode side is smaller and the number of pores is larger, and the pore size on the cathode side is larger and the number is smaller. This is consistent with our model predictions. In addition, the previous electroporation experiments conducted by our research group^22^ also verify the pore density and pore radius distribution predicted by the model.

### B. Effect of buffer osmotic pressure on cell electrofusion

#### 1. Effect of cell membrane tension on electrofusion efficiency

As mentioned previously, hypotonic buffer facilitates electroporation and electrofusion of lipid bilayers and mammalian cells at a lower electric field intensity. Meanwhile, studies have shown that hypotonic buffer increases cell membrane tension by inducing osmotic swelling of cells compared with isotonic and hypertonic buffers.^25,26^ Mukherjee *et al.*^28^ calculated that the higher membrane tension induced by the hypotonic buffer is beneficial to the local electroporation of cells. They verified the mathematical simulation results through experiments. On the basis of Mukherjee, we further predicted that hypotonic buffer can improve the electrofusion efficiency of cells by inducing a higher membrane tension compared with isotonic and hypertonic buffers. To verify the above-mentioned prediction, we applied the established model to study the effect of membrane tension induced by different osmotic pressures on cell electrofusion. Three different values of membrane tension, 30, 150, and 750 pN/*μ*m, were used.^29^ They corresponded to the hypertonic (360 mOsmol/kg), isotonic (280 mOsmol/kg), and hypotonic buffers (200 mOsmol/kg) used in subsequent electrofusion experiments. It is important to note that the membrane tension mentioned in this section is the initial tension ( $\sigma_{0}$) of the non-electroporated cell membrane.

**FIG. 2.**
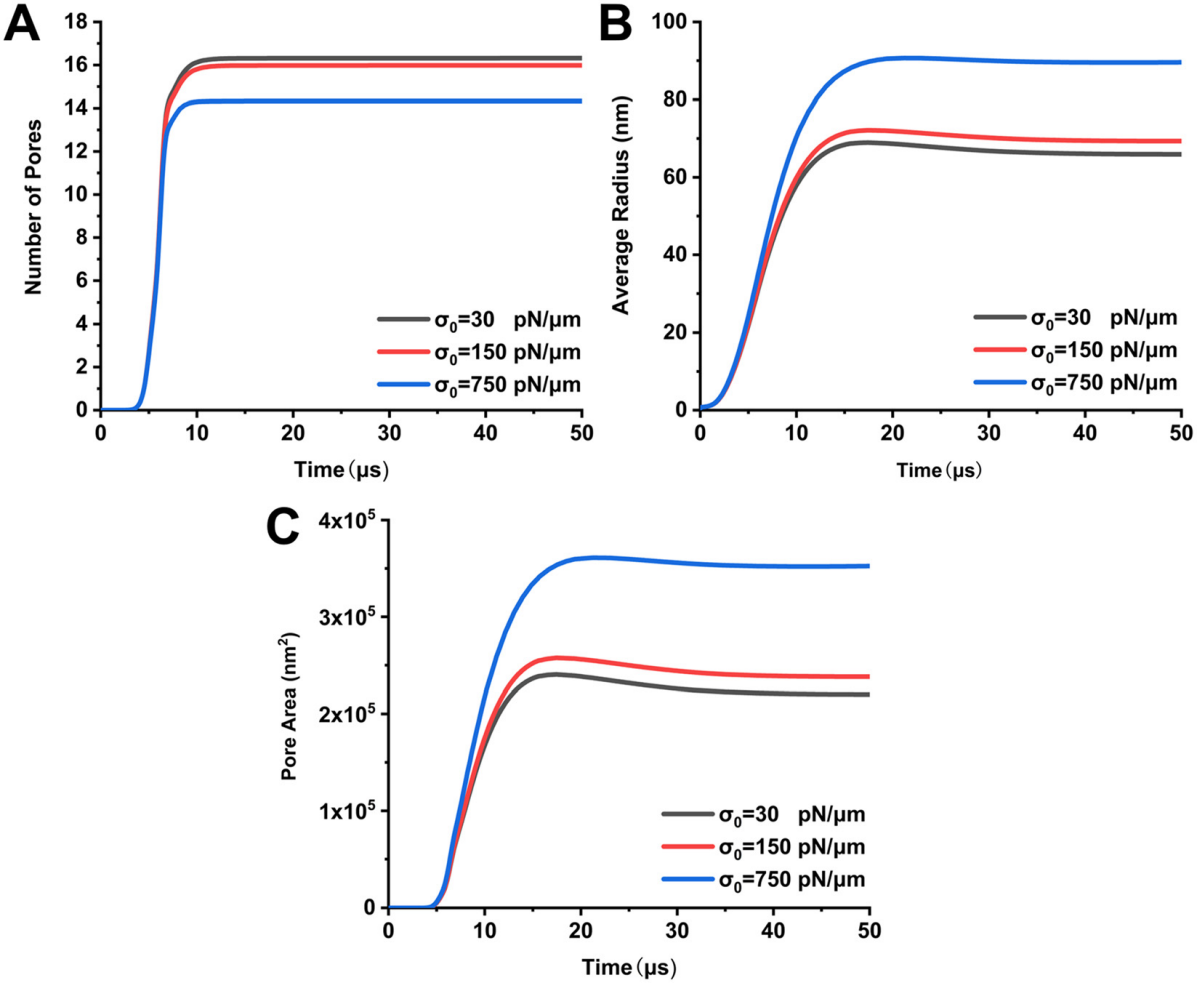
Time-dependent plot of electroporation in the cell-cell contact zone during electrical pulses at different values of membrane tension. (a) The number of pores; (b) the average radius of pores; and (c) the area of pores.

We first calculated the number of pores [Fig. 2(a)] and the average radius of pores [Fig. 2(b)] produced in the cell–cell contact zone at three membrane tension values. The results show that there is little difference in the number of pores and the average radius of pores for membrane tension (30 pN/*μ*m and 150 pN/m) induced by hypertonic and isotonic buffers. When the membrane tension is increased to 750 pN/*μ*m induced by the hypotonic buffer, the number of pores decreases slightly from 16 (30 pN/*μ*m and 150 pN/m) to 14, but the average radius of pores expands significantly from about 66 nm (30 pN/*μ*m and 150 pN/m) to 90 nm. This suggests that as the membrane tension increases, fewer but larger pores are created in the cell–cell contact zone. The decrease in the number of pores in the cell–cell contact zone weakened the cytoplasm exchange between the two cells, but the expansion of the pore radius enhanced the above-mentioned phenomenon. We cannot determine whether the increased membrane tension in the hypotonic buffer facilitates the cytoplasm exchange during cell electrofusion based only on the number of pores and pore radius. Therefore, we further calculated the effect of membrane tension on the area of all pores formed in the cell–cell contact zone [Fig. 2(c)]. The pore area of the cell–cell contact zone is a direct determinant of cytoplasm exchange between two cells, which can more intuitively illustrate the effect of membrane tension on cell electrofusion. We also found there is no significant difference in pore area when the membrane tension is 30 pN/*μ*m or 150 pN/*μ*m, which is about 2.2 × 10^5^ nm^2^. When the membrane tension increases to 750 pN/*μ*m, the pore area increases to 3.6 × 10^5^ nm^2^, an increase of about 1.7 times. Larger pore area means that subsequent cytoplasm exchange between cells will be easier, which is beneficial for cell electrofusion. These results suggest the hypotonic buffer is able to increase the reversible electroporation area in the cell–cell contact zone by inducing a higher membrane tension compared to hypertonic and isotonic buffers, which facilitate subsequent cytoplasm exchange and cell fusion.

#### 2. Effect of cell size on electrofusion efficiency

**FIG. 3.**
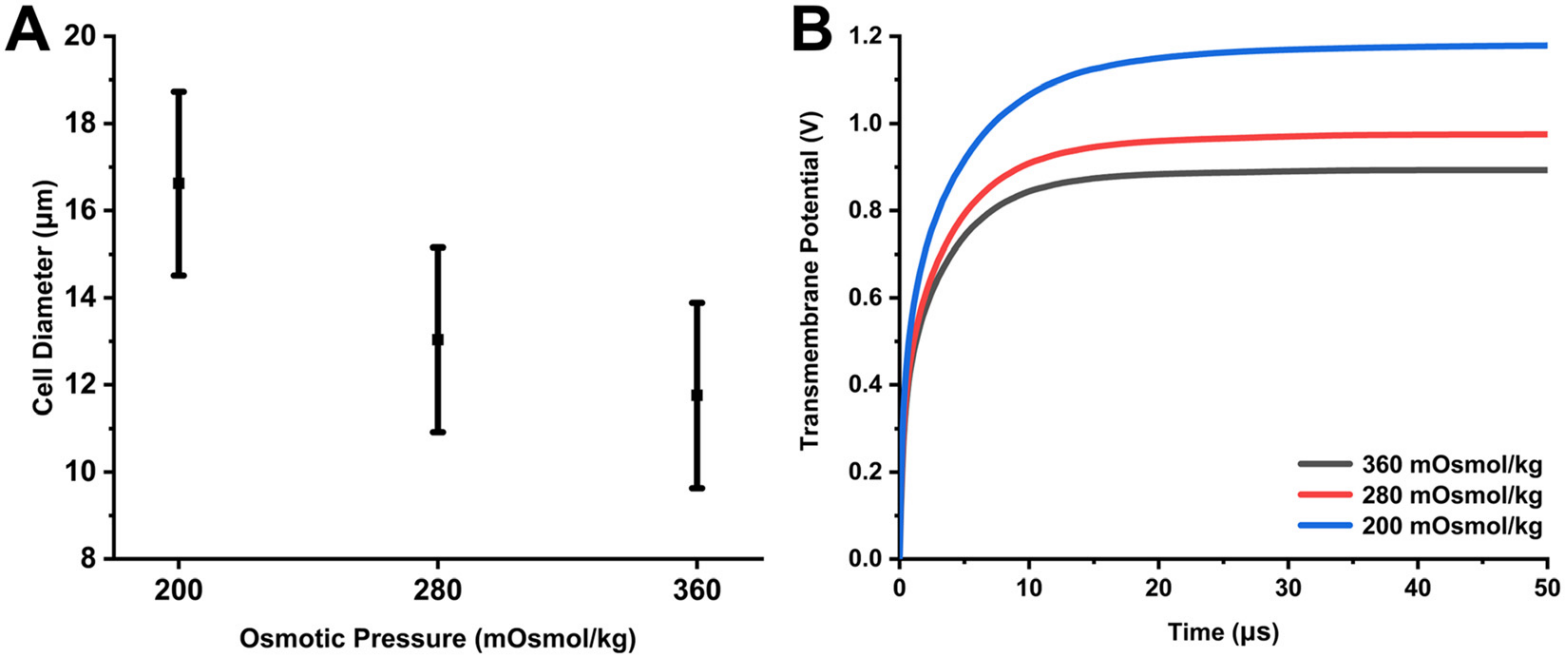
(a) Cell size under different osmotic pressure buffers and (b) the TMP changes at the P_2_ point in hypertonic, isotonic, and hypotonic buffer at the same applied voltage.

In addition to affecting electrofusion efficiency through membrane tension itself, osmotic pressure buffers can also alter cell size through membrane tension, and cell size determines the TMP. We counted the size of SP2/0 cells in hypertonic, isotonic, and hypotonic buffers. In previous cell size counting methods, one osmotic pressure buffer corresponds to a batch of cells.^26,30^ In our experiments, only one batch of cells was used and the cell sizes in the three osmotic pressure buffers were counted by buffer replacement. This method can effectively reduce the random error between different batches of cells and improve the accuracy of statistical results. Supplementary material, Fig. S1 shows the cell size of SP2/0 cells in hypertonic, isotonic, and hypotonic buffers, respectively. Cell size is generally larger in hypotonic buffers, followed by isotonic buffers, and smallest in hypertonic buffers. Figure 3(a) shows the statistical results of cell size in the three osmotic pressure buffers. The cell diameters in hypertonic, isotonic, and hypotonic buffers are 11.76 ± 2.13 *μ*m, 13.04 ± 2.12 *μ*m, and 16.62 ± 2.11 *μ*m, respectively. Based on the cell size, we calculated the TMP at the P_2_ point [Fig. 3(b)]. In the hypotonic buffer, the TMP at the P_2_ point reaches a maximum value of 1.17 V after reaching a steady state. In the isotonic buffer, the TMP is the second. In the hypertonic buffer, the TMP is the smallest at 0.89 V. The TMP increased significantly in the hypotonic buffer compared to hypertonic and isotonic buffers. This means that the applied voltage required for cells to reach the threshold electroporation voltage in hypotonic buffer is significantly lower than that in hypertonic and isotonic buffers. Therefore, cells in a hypotonic buffer are more likely to be electrofused.

### C. Experiment verification of electrofusion

**FIG. 4.**
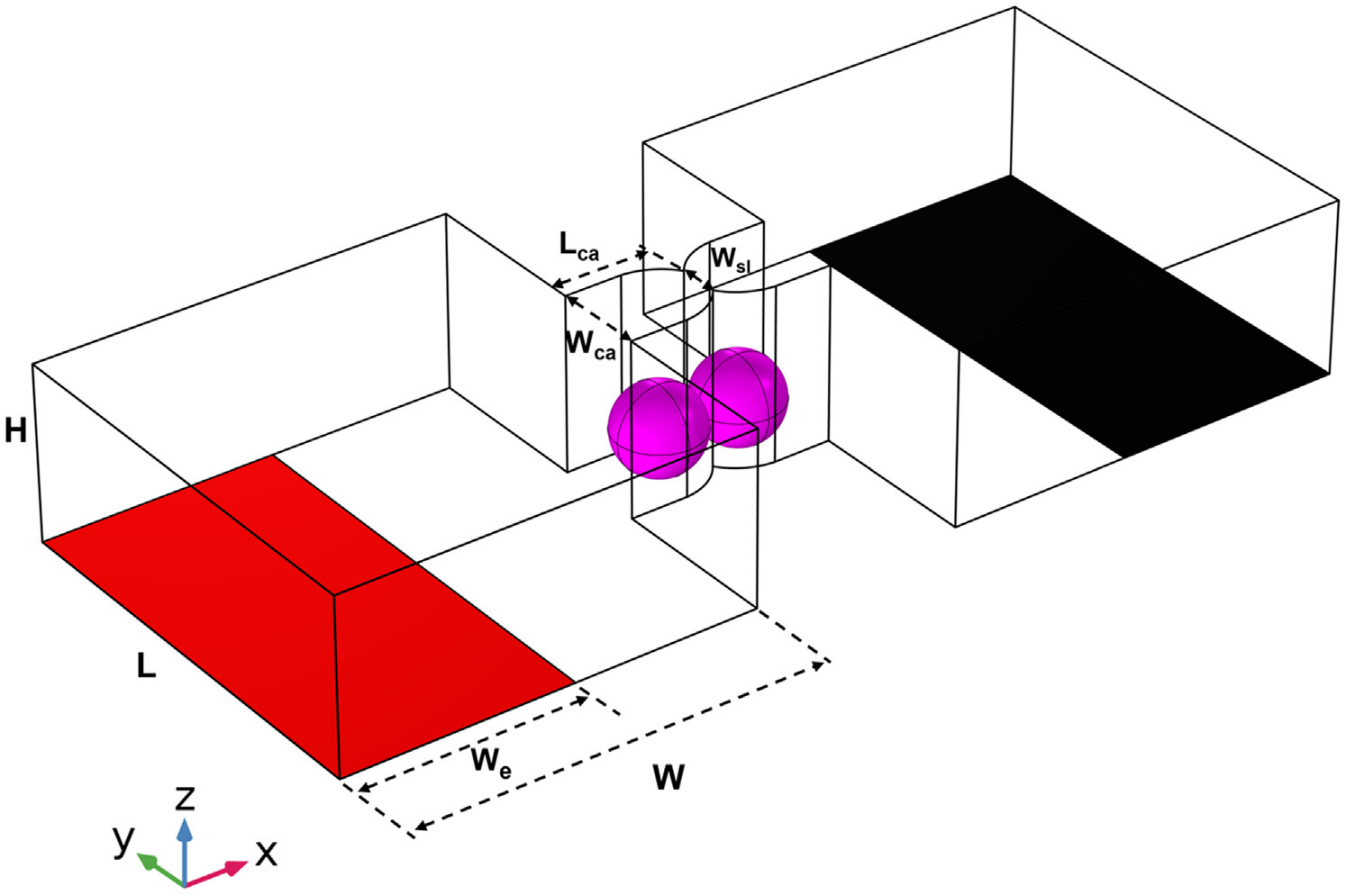
Microchannel used for cell electrofusion.

**FIG. 5.**
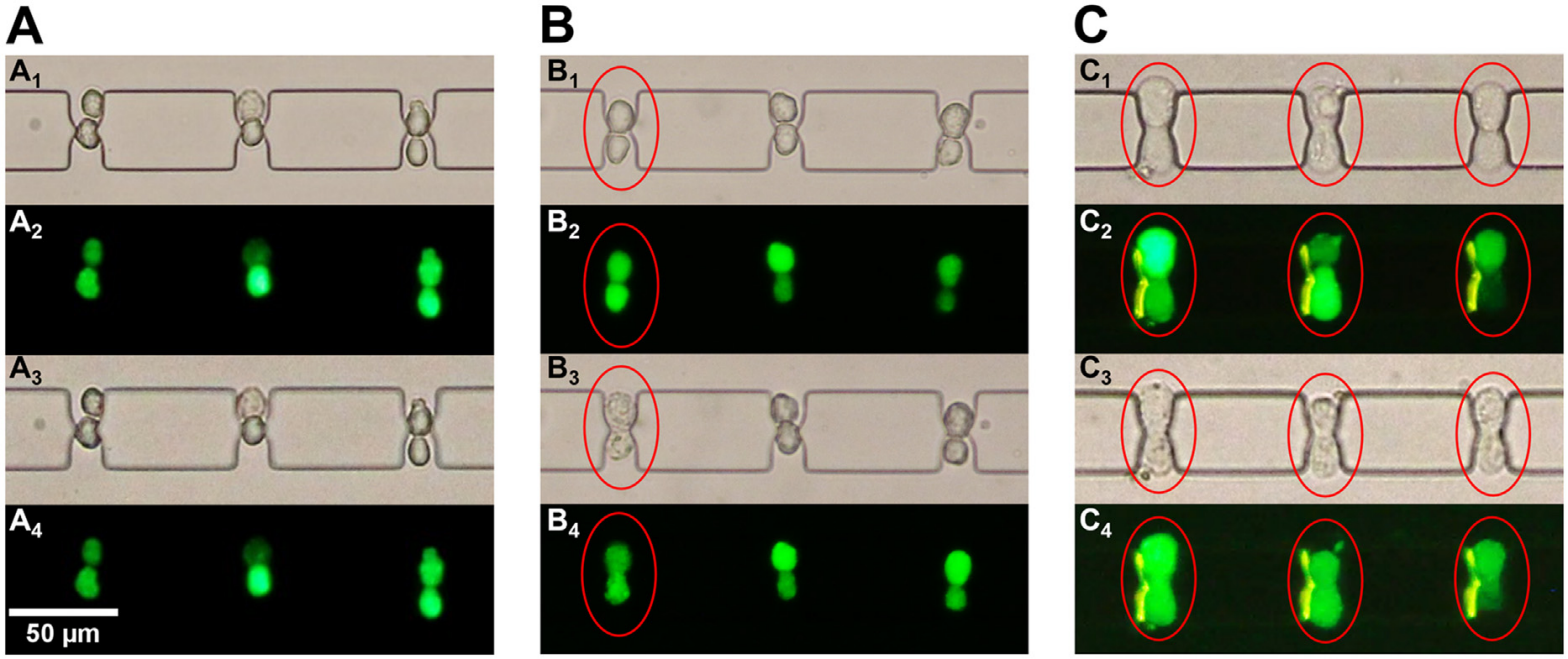
Cell fusion results in different osmotic pressure buffers. (a) hypertonic buffer; (b) isotonic buffer; and (c) hypotonic buffer. A_1_, B_1_, and C_1_ are bright field images of the cell state before applying DC pulses (*t* = 0). A_2_, B_2_, and C_2_ are fluorescence images of the cell state before applying DC pulses (*t* = 0). A_3_, B_3_, and C_3_ are the bright field images of the cell state after applying the DC pulses for 5 min (*t* = 5 min). A_4_, B_4_, and C_4_ are the fluorescence images of the cell state after applying the DC pulses for 5 min (*t* = 5 min).

To verify the above-mentioned theoretical predictions, we used SP2/0 cells and the electrofusion microstructure shown in Fig. 4 to perform cell electrofusion under hypertonic (360 mOsmol/kg), isotonic (280 mOsmol/kg), and hypotonic (200 mOsmol/kg) buffer, respectively. Figure 5 depicts bright field and fluorescence images of three pairs of cells during the fusion steps. The cells marked by the red oval line are the fused cells. First, the fluorescence is confined to paired cells and does not leak into the buffer, indicating that no cell rupture occurs during this process. Second, the fluorescence intensity within the cells on one side is reduced because their fluorescence is transferred to the cells on the other side through the pores formed in the cell–cell contact zone. We calculated the fusion efficiency of the 12 pairs of cells after pairing was completed (supplementary material, Fig. S2). In the hypertonic buffer, the intracellular fluorescence intensity remained almost constant before and after the application of DC pulses of up to 15 V, which means that the cells did not undergo electroporation and electrofusion. In the isotonic buffer, cell fusion occurred when DC pulses of 12 V were applied, but the cell fusion efficiency was only 7%. In the hypotonic buffer, the cell fusion efficiency was as high as 60% when DC pulses of 10 V were applied. The cell fusion efficiency was significantly higher, and the applied voltage was lower in hypotonic buffers compared to hypertonic and isotonic buffers. The experimental results are qualitatively consistent with the above-mentioned theoretical predictions.

In addition, we also found an interesting phenomenon in the electrofusion experiments and *in situ* cell size counting. The cells were mostly spherical in hypotonic buffer and ellipsoidal in isotonic and hypertonic buffers. We believe that this change in the cell morphology also affects the electrofusion efficiency. For ellipsoidal cells in isotonic and hyperosmotic buffers, a cell–cell contact zone tends to form at the long-axis end when the two cells are paired under DEP force. This is because the curvature at the cell long-axis end is greater than those at other locations, and the electric field intensity at that location is higher. House's study on the final orientation of the two ellipsoidal particles under electrophoretic forces is consistent with our above-mentioned viewpoint.^31^ This pairing of elliptical cells has two effects. First, the cell–cell contact zone is smaller, and therefore, the reversible electroporation area is reduced. Second, the paired ellipsoidal cells have poor stability during the electrofusion process due to the high curvature and electric field intensity at the long-axis end.^32^ When a series of DC pulses are applied to induce cell reversible electroporation, the cells are prone to slip or rotate due to poor stability. Even if two cells undergo electroporation at the cell–cell contact zone, the position of the pores changes as the cell slides or rotates, which prevents subsequent membrane reconstruction and cytoplasm exchange. Both of these factors reduce the electrofusion efficiency. Cells are mostly spherical in hypotonic buffer, and the cell–cell contact zone is significantly increased in this morphology compared to ellipsoidal cells in isotonic and hypertonic buffers. The reversible electroporation area in the cell–cell contact zone also increases accordingly when electroporation occurs. The above-mentioned processes work together with a higher membrane tension to improve the cell fusion efficiency in hypotonic buffers.

## III. CONCLUSION

In order to explore the mechanism by which buffer osmotic pressure influences cell electrofusion, we developed an electroporation model describing the cell electrofusion process in microchannels with paired arc micro-cavities. The model was further validated based on reported experiments. Using the developed model, we found the hypotonic buffer improves cell electrofusion efficiency by inducing a higher membrane tension and larger cell size. In the subsequent electrofusion experiments, we verified the above-mentioned prediction. Various properties of electroporation and electrofusion buffers, such as osmotic pressure, conductivity, and ion concentration, as well as electroporation and electrofusion microstructure parameters, can be modified and optimized using our proposed modeling tool prior to experimental testing. Our model tools can also be used for other applications of electroporation, such as cell electrotransfection, lysis, and tumor ablation.

(1) The microfluidic structure with paired arc micro-cavities can selectively control the location of electroporation in the cell–cell contact zone, which is ideal for cell electrofusion. The pore density in this zone is as high as 7.91 × 10^11^ m^−2^, and the pore size is moderate with a radius of about 60 nm. Although the two cells are located in symmetrical positions in the microchannel, their pore distribution is asymmetrical due to the resting potential.
(2) Hypotonic buffers increase the reversible electroporation area in the cell–cell contact zone by 1.7 times by inducing a higher membrane tension compared to lower membrane tension in hypertonic and isotonic buffers, which facilitates subsequent cytoplasm exchange and cell fusion. The increased membrane tension in the hypotonic buffer further enlarges the cell size, which reduces the applied voltage required for cell electroporation and electrofusion and causes less damage to the cells.
(3) No cell electroporation or electrofusion occurred with the application of pulses of up to 15 V in hypertonic buffer. The voltage required for cell electroporation and electrofusion was 12 V in isotonic buffer, and the cell fusion efficiency was only 7%. In the hypotonic buffer, the applied voltage was 10 V, and the cell fusion efficiency was 60%.

## IV. METHODS

### A. Numerical model

Figure 4 shows the microfluidic structure used in the cell electrofusion study. This structure is improved based on the study of Murat *et al.*^33^ In order to enhance the electric field intensity inside the micro-cavities and reduce cell damage, we change the trapezoidal micro-cavities into arc micro-cavities. This is because, when comparing the two types of micro-cavities (Fig. S3), it can be observed that the arc micro-cavity shows a higher intensity of the electric field, with the electric field predominantly concentrated at the surface of cell–cell contact. On the other hand, the electric field strength at the non-contact location between cells is considerably lower, thereby minimizing cell damage. Considering the size of cells in electrofusion experiments, the size of the arc micro-cavities is set as *L_ca_* × *W_ca_* and the width of the micro-slit between a pair of micro-cavities is *W_sl_*. On both sides of the micro-cavities, we consider two parallel main channels of length *L* and width *W*. The height of the microchannel consisting of the micro-cavities and the main channels is *H*, and it is filled with a buffer of conductivity $\sigma_{o}$. Spherical cells of radius *a* are suspended in the buffer. In order to make the paired cells not completely continuous, we set the distance between cells is 0.001 *μ*m. The red and black surfaces in Fig. 4 represent the microelectrodes, which are located on the bottom of the microchannel. Cells in the left and right main channels are paired in the micro-cavities under the action of positive dielectrophoretic force.

The potentials of intracellular $\emptyset_{i}$ and extracellular $\emptyset_{o}$ can be calculated using the following electrostatic equations:^34^

$$-\nabla \left(\sigma_{i}\nabla \emptyset_{i}\right)-\varepsilon_{0}\varepsilon_{i}\nabla \left(\frac{\partial }{\partial t}\left(\nabla \emptyset_{i}\right)\right)=0,$$ (1)

$$\text{and}\;-\nabla \left(\sigma_{o}\nabla \emptyset_{o}\right)-\varepsilon_{0}\varepsilon_{o}\nabla \left(\frac{\partial }{\partial t}\left(\nabla \emptyset_{o}\right)\right)=0,$$ (2)

where $\varepsilon_{0}$ is the dielectric permittivity of vacuum. $\varepsilon_{i}$, $\sigma_{i}$ and $\varepsilon_{o}$, $\sigma_{o}$ are the relative permittivity and conductivity of cytoplasm and electrofusion buffer, respectively.

The transmembrane potential (TMP) is defined as

$$\Delta \emptyset \left(t\right)=\emptyset_{i}\left(t\right)-\emptyset_{o}\left(t\right).$$ (3)

$\Delta \emptyset \left(0\right)=V_{\text{rest}}$ is the resting potential. When cells are not exposed to an external electric field, there are certain number of ions on the inner and outer surfaces of the cell membrane. Since the ion channels and carrier proteins embedded in the lipid bilayer allow ions to pass through the cell membrane, the ion concentration inside and outside the cell membrane is different, resulting in the resting potential.^35^

The voltage ( $\emptyset_{0}$) required for electrofusion was applied via electrodes at the bottom of the microchannel. Therefore, the boundary condition on the electrodes is an electric potential ( $\emptyset_{0}$ on red surface) or grounded (on black surface). An electrical insulation boundary condition is applied on the remaining walls of the microchannel. Cell membrane is not physically included in the model. This is because the thickness of the cell membrane is about 5 nm, which is much smaller than the cell radius (7.5 *μ*m). Including cell membrane in the model would be computationally expensive. To represent the cell membrane, the current density flowing through the cell membrane is used,^36^

$$J\left(t\right)=\frac{\sigma_{m0}\Delta \emptyset (t)}{d_{m}}+\frac{\varepsilon_{0}\varepsilon_{m}}{d_{m}}\frac{\partial \Delta \emptyset (t)}{\partial t}+I_{p}(t).$$ (4)

For non-electroporated cells, the current density across the cell membrane only includes the first and second terms on the right side of Eq. (4). They represent the conductance and capacitance components across the cell membrane, respectively. $\sigma_{m0}$ is the non-electroporated cell membrane conductivity; $\varepsilon_{m}$ and $d_{m}$ are the relative permittivity and thickness of the cell membrane, respectively. When an appropriate electric field is applied, cells will undergo electroporation. For electroporated cells, the current density changes due to the creation of nanopores.^36^ In addition, $I_{p}(t)$ is the current density through the pores, which is calculated by^36^

$$I_{p}(t)=N\left(t\right)i_{p}\left(r_{p},\Delta \emptyset (t)\right),$$ (5)

where $i_{p}$ is the current through a nanopore, which is a function of the pore radius $r_{p}$ and TMP. The pores density *N* can be obtained from the following differential equation:^34–36^

$$\frac{dN\left(t\right)}{dt}=\alpha e^{{\left(\Delta \emptyset (t)/V_{\mathrm{EP}}\right)}^{2}}\left(1-\frac{N\left(t\right)}{N_{0}}e^{{-q\left(\Delta \emptyset (t)/V_{\mathrm{EP}}\right)}^{2}}\right),$$ (6)

where *N*_0_ is the equilibrium pore density at $\Delta \emptyset \left(t\right)=0$. The parameters α, q, and $V_{\mathrm{EP}}$ characterize the electroporation process.

For a formed nanopore with radius $r_{p}$, the current through it is^35^

$$i_{P}\left(r_{p},\Delta \emptyset (t)\right)=\frac{\Delta \emptyset (t)}{R_{p}+R_{i}}=\frac{\Delta \emptyset (t)}{\frac{d_{m}}{\pi \sigma_{p}r_{p}^{2}A}+\frac{1}{2\sigma_{p}r_{p}}},$$ (7)

where $R_{p}$ is the pore resistance, $R_{i}$ is the input resistance,^35,36^ and $\sigma_{p}$ is the conductivity of the solution filling the pore. The parameter *A* in Eq. (7) is defined as^36^

$$A=\frac{e^{v_{m}}-1}{e^{v_{m}}(w_{0}e^{{w_{0}-nv}_{m}}-{nv}_{m})/{{(w}_{0}-nv}_{m})-(w_{0}e^{{w_{0}+nv}_{m}}+{nv}_{m})/{{(w}_{0}+nv}_{m})},$$ (8)

where n is the relative entrance length of the pore and $w_{0}$ is the energy barrier inside the pore. $v_{m}=\Delta \emptyset (t)F/RT$. *F*, *R*, and *T* are the Faraday constant, the gas constant, and the absolute temperature of the buffer, respectively. The initial pores with radius $r^{*}$ change size to minimize the energy of the lipid bilayer. The change rate of its radius is governed by the following ordinary differential equation:^35^

$$\frac{dr_{p}}{dt}=\frac{D}{kT}\left\{\frac{{\Delta \emptyset (t)}^{2}F_{\text{max}}}{1+\frac{r_{h}}{r_{p}+r_{t}}}+{4\beta (\frac{r^{*}}{r_{p}})}^{4}\frac{1}{r_{p}}-2\pi \gamma +2\pi \sigma_{\text{eff}}r_{p}\right\}\;{(r}_{p}\geq r^{*},p=1,2,\ldots ,n),$$ (9)

where *D* represents the pore radius diffusion coefficient and *k* is the Boltzmann constant. The four terms on the right-hand side of Eq. (9) represent, in order, the electrodynamic force induced by the local TMP, the steric repulsion of the lipid heads, the line tension acting on the pore perimeter, and the cell membrane tension, respectively. $\sigma_{\text{eff}}$ is the effective membrane tension, which is a function of $A_{p}$. $A_{p}=\sum_{p=1}^{n}\pi r_{p}^{2}$ represents the sum of the areas of all pores in the cell membrane.^35^ $\sigma_{\text{eff}}$ is calculated by the following formulas, respectively:

$$\sigma_{\text{eff}}={2\sigma }^{\prime }-\frac{{2\sigma }^{\prime }-\sigma_{0}}{{(1-A_{p}/A_{c)}}^{2}},$$ (10)

where $\sigma_{0}$ is the membrane tension of the non-electroporated cell; $\sigma^{\prime }$ is the energy per unit area of the hydrocarbon–water interface; and $A_{c}$ is the surface area of the cell.

To solve the above-mentioned equations, the finite element software COMSOL Multiphysics was used. The distribution of the electric field within the microchannels and the potentials inside and outside the cells were calculated using two current modules in the AC/DC physical field. In each current module, normal current density boundary conditions were added on the cell membrane separately, and Eqs. (4) and (5) were applied to the cell membrane to control the change in current density flowing through the cell membrane before and after electroporation. To numerically simulate the density of cell membrane pores, a PDE module with a general form partial differential equation boundary condition was added. Equations (6)–(8) were applied to the cell membrane to study the electroporation density under the influence of the electric field. Meanwhile, in the general form partial differential equation boundary condition, Eqs. (9) and (10) were applied to the cell membrane to control the radius of electroporation. The parameters required in the solution process are listed in Table I.

**TABLE I.** Constants and parameter values used in the simulation.^38^

Parameters	Description	Value
*H* × *W* × *L*	Main channel size	30 × 72 × 76 *μ*m^3^
$L_{ca}$× $W_{ca}$	Arc micro-cavity size	18 × 16 *μ*m^2^
$W_{e}$	Microelectrode width	40 *μ*m
$W_{sl}$	Micro-slit width	7 *μ*m
*a*	Cell radius	7.5 *μ*m
$\sigma_{o}$	Buffer conductivity	0.0053 S m^−1^
$\varepsilon_{o}$	Buffer relative permittivity^34^	80
$\sigma_{i}$	Cytoplasm conductivity^34^	0.3 S m^−1^
$\varepsilon_{i}$	Cytoplasm relative permittivity^6^	70
$d_{m}$	Cell membrane thickness^34^	5 nm
$\sigma_{m0}$	Cell membrane conductivity^37^	9.5 × 10^–9^ S m^−1^
$\varepsilon_{m}$	Cell membrane relative permittivity^6^	4.5
$\sigma_{p}$	Conductivity of the solution inside the pore^38^	$\frac{\sigma_{o}-\sigma_{i}}{\text{log}(\sigma_{o}-\sigma_{i})}$
$V_{\text{rest}}$	Resting potential^35^	−50 mV
*N*_0_	Equilibrium pore density at Δ∅=0^34^	1.5 × 10^9^ m^−2^
α	Creation rate coefficient of pore^34^	1 × 10^9^ m^−2^ s^−1^
*q*	Creation rate constant of pore^38^	${\left(\frac{r_{m}}{r^{*}}\right)}^{2}$
*V_EP_*	Characteristic voltage of electroporation^36^	258 mV
*n*	Relative entry length of the pore^36^	0.15
*w*_0_	Energy Barrier in the pore^36^	2.65
*F*	Faraday constant	9.65 × 10^4^ C mol^−1^
*R*	Gas constant	8.314 J K^−1 ^mol^−1^
*T*	Absolute temperature	295 K
*D*	Diffusion coefficient of pore radius^35^	5 × 10^–14^ m^2^ s^−1^
*k*	Boltzmann constant	1.3806 × 10^–23^ J K^−1^
$F_{\text{max}}$	Maximum power at Δ∅=1 V^35^	0.7 × 10^–9^ N V^−2^
β	Steric repulsion energy^35^	1.4 × 10^–19^ J
γ	Edge energy^35^	1.8 × 10^–11^ J m^−1^
$r^{*}$	Minimum radius of hydrophilic pore^35^	0.51 nm
$r_{m}$	Minimum energy radius of hydrophilic pore^35^	0.8 nm
$\sigma_{0}$	Membrane tension of non-electroporated cell^35^	1 × 10^–6^ J m^−2^
$\sigma^{\prime }$	Tension of hydrocarbon-water interface^35^	2 × 10^–2^ J m^−2^
$r_{h}$	Electricity constant of characteristic length^35^	0.97 nm
$r_{t}$	Electricity constant for pore correction^35^	0.31 nm

### B. Cell culture and cell preparation

Sp2/0 cells (Chongqing Academy of Animal Sciences, Chongqing, China) labeled by green fluorescence (CytoTrace Green CMFDA) were used to monitor the electrofusion process. They were cultured in Roswell Park Memorial Institute (RPMI) 1640 supplemented with 10% Fetal Calf Serum (FCS) and 1% penicillin/streptomycin, and incubated in a humid environment at 37 °C containing 5% CO_2_ and 95% air. Prior to experiments, cells were digested with pronase (0.5 mg/ml, Shanghai yuanye Bio-Technology Co., Ltd, Shanghai, China) and collected. After collecting, the cells were washed three times with electrofusion buffer (osmotic pressure: 200, 280, and 360 mOsmol/kg and conductivity: 53 *μ*S/cm, Eppendorf Co., Eukaryotic Cell Electrofusion Buffer, Germany), and suspended as a concentration of 2.0 × 10^6^ cells/ml for cell electrofusion.

### C. Experiments protocol

Microfluidic chip is composed of microelectrode layer and polydimethylsiloxane (PDMS) (Sylgard 184, Dow Corning, Midland, MI, USA) microchannel layer. The microchannel with arrayed arc micro-cavities was designed based on numerical simulation and fabricated by transferring microchannel patterns onto PDMS using a mold.^21,39^ Electrode was fabricated by indium tin oxide (ITO) (100-nm ITO film thickness, 17 Ω/sq.) because of its good transparency. The distance between the electrodes was 100 *μ*m, and the electrodes were located on both sides of paired arc micro-cavities. Then, an O_2_-plasma (150 W, 60 s) activation (Shenzhen Fangrui Technology Co., Ltd., Shenzhen, China) was performed on the ITO and PDMS surfaces to reversibly bind the PDMS to the ITO. The fabricated microfluidic chip was packaged to connect with an external cell electrofusion machine for experiments.

Before the experiment, the microfluidic chip was rinsed with electrofusion buffer for 10 s. The SP2/0 cell suspension was pipetted into the microchannel at a flow rate of 20 *μ*l/h. The AC pulses (amplitude: 3 V, frequency: 1 MHz, and duration: 30 s) were then applied to the microelectrodes. The cells were attracted to the paired arc micro-cavities by the positive dielectrophoretic (DEP) force to complete cell pairing.^40^ Finally, a series of direct current (DC) electric pulses (amplitude: 12 V, duration: 50 *μ*s, interval between two pulses: 1 s, and pulses number: 5) were applied to induce cell fusion. The whole process was monitored by an Olympus BX 61 microscope (Olympus Optical Co. Ltd., Japan) with a Canon camera (EOS60D, Canon Co., Ltd. Japan).

## SUPPLEMENTARY MATERIAL

See the supplementary material for additional experimental results.

## Data Availability

The data that support the findings of this study are available from the corresponding authors upon reasonable request.
